# BayesMotif: de novo protein sorting motif discovery from impure datasets

**DOI:** 10.1186/1471-2105-11-S1-S66

**Published:** 2010-01-18

**Authors:** Jianjun Hu, Fan Zhang

**Affiliations:** 1 Department of Computer Science and Engineering, University of South Carolina, Columbia, SC, 29208, USA

## Abstract

**Background:**

Protein sorting is the process that newly synthesized proteins are transported to their target locations within or outside of the cell. This process is precisely regulated by protein sorting signals in different forms. A major category of sorting signals are amino acid sub-sequences usually located at the N-terminals or C-terminals of protein sequences. Genome-wide experimental identification of protein sorting signals is extremely time-consuming and costly. Effective computational algorithms for de novo discovery of protein sorting signals is needed to improve the understanding of protein sorting mechanisms.

**Methods:**

We formulated the protein sorting motif discovery problem as a classification problem and proposed a Bayesian classifier based algorithm (BayesMotif) for de novo identification of a common type of protein sorting motifs in which a highly conserved anchor is present along with a less conserved motif regions. A false positive removal procedure is developed to iteratively remove sequences that are unlikely to contain true motifs so that the algorithm can identify motifs from impure input sequences.

**Results:**

Experiments on both implanted motif datasets and real-world datasets showed that the enhanced BayesMotif algorithm can identify anchored sorting motifs from pure or impure protein sequence dataset. It also shows that the false positive removal procedure can help to identify true motifs even when there is only 20% of the input sequences containing true motif instances.

**Conclusion:**

We proposed BayesMotif, a novel Bayesian classification based algorithm for de novo discovery of a special category of anchored protein sorting motifs from impure datasets. Compared to conventional motif discovery algorithms such as MEME, our algorithm can find less-conserved motifs with short highly conserved anchors. Our algorithm also has the advantage of easy incorporation of additional meta-sequence features such as hydrophobicity or charge of the motifs which may help to overcome the limitations of PWM (position weight matrix) motif model.

## Background

A typical cell has a size of only 10 μm while it contains about a billion proteins. How these proteins are transported from their synthesis sites to their target locations within or outside of the cell is still not well understood. Experiments showed that translocation of nascent proteins are usually guided by "postal code" like targeting signals encoded within the amino acid sequences of proteins. Genome-wide identification and decoding of these molecular "zip codes" are fundamental to the understanding of the cell. Experimentally identifying protein targeting signals is labor and cost intensive, usually using a tedious cut-and-test approach [1,2]. Recently, genome scale protein localization data has become available [3] for a couple of species and gene ontology also provides a large amount of localization information of proteins [4]. These datasets provide a great opportunity for developing bioinformatic algorithms to identify protein sorting signals to guide biological experiments.

However, computational prediction of targeting signals is still a big challenge due to their low conservation at the amino acid level. Many motif discovery algorithms [5] have been proposed in the past decades but mostly have been only tested or applicable to DNA motif discovery with alphabet of four nucleotides rather than 20 amino acids. These de novo motif discovery algorithms such as MEME [6] and TEIRESAS [7] are not very effective to mine protein sorting signals due to their low conservation at the amino acid level in terms of variation of their composition and motif widths. Many algorithms such as targetP [8] and Bacello [9] have been developed recently for predicting subcellular localization from protein sequences. However these algorithms cannot identify sorting signals. The most well-known protein sorting motif prediction algorithm SignalP [10] is widely used to predict the presence and location of signal peptide cleavage sites in amino acid sequences. But it is built on the well-known secretory sorting motif model and lacks the capability of de novo motif discovery algorithms to identify novel sorting motif models. There are several web servers for funcational site annotation for protein sequences such as the Eukaryotic Linear Motif (ELM) server [11]. However most of these servers are limited to regular expression based pattern scanning with known motif models. There is no good tool for de novo identification of sorting motifs from a given set of protein sequences which are sorted to the same subcellular location.

In this paper, we are interested in algorithms for de novo discovery of a common type of protein sorting motifs that are composed of a highly conserved anchor (2 to 5 amino acids long) and a less conserved amino acid region with specific physichemical properties. Most of these sorting signals are located within the 200 amino acids of the N-terminal or C-terminal of the protein sequence. For example, Chaddock et al. [1] examined thylakoid transfer signals from all of the known lumenal proteins and found that all of the substrates for the ApH-dependent translocase possess a twin-arginine motif (RR) immediately before the hydrophobic (H) amino acid region. Brink [12] showed that the RR motif alone is not sufficient for the delta pH transportation and another signal inside the hydrophobic region is required. Sheikh and Isacke reported a di-hydrophobic motif Leu330-Val334 motif which is located within a cytoploasmic domain [13].

**Figure 1:**
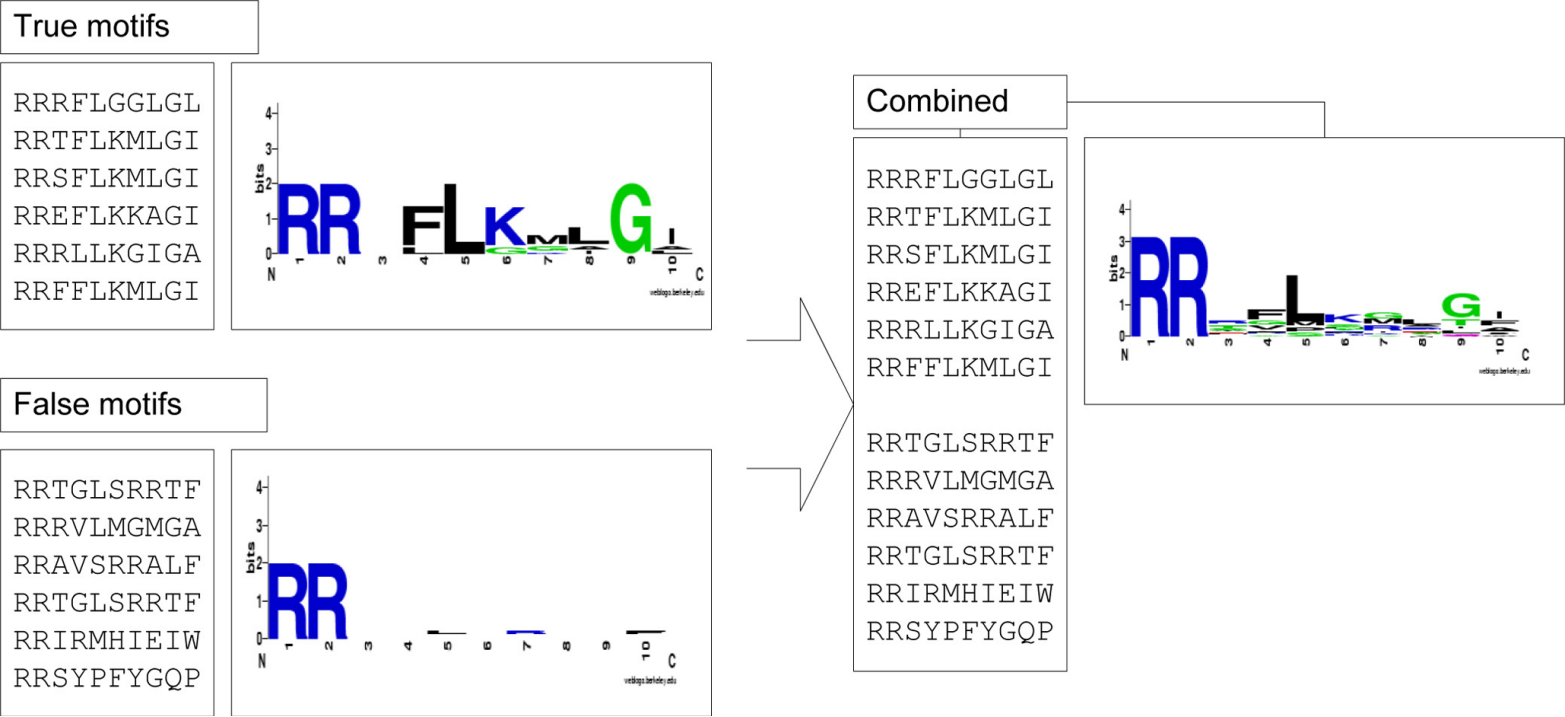
**Mixture of true signal motifs with false motifs severely reduced the conservation level of the motif regions**.

Recently, we proposed a Bayesian classifier based algorithm [14] for de novo discovery of protein sorting motifs with anchors. The key idea is to scan the neighbourhood of over-represented anchors on the protein sequences to identify those motifs that can differentiate positive sequences which are expected to contain sorting motifs to negative background sequences which are supposed to contain no motifs. This algorithm was shown to be able to retrieve both implanted motifs from benchmark data and real sorting motifs from real-world datasets. However, it is observed that this algorithm failed to find sorting motifs if the percentage of sequences with true motif instances in the positive training dataset drops to a certain low-level (e.g. <50%), when the classification accuracy of the underlying Bayesian classifier cannot differentiate the positive dataset from the negative dataset and thus cannot identify correct motifs. As shown in Figure 1, when the true positive datasets are mixed with false positive dataset, the conservation level of the sorting signals become weak. It is thus necessary to detect false positive sequences since it is difficult to prepare pure positive sequence sets as there are usually multiple pathways targeting to a specific subcellular location.

In this paper, we introduced a false positive removal mechanism to handle the false positive sequence issue. Our enhanced BayesMotif algorithm is shown to be able to find true sorting motifs even when the percentage of the true positive sequences is less than 10% on our benchmark datasets or less than 20 on real-world datasets. This function is similar to traditional motif discovery algorithm such as MEME which can allow sequences without motifs in the input dataset.

## Methods

### Overview of the BayesMotif algorithm

We formulate the protein sorting motif discovery problem as a classification problem: Given a set of protein sequences *P *= {*s*_1_, *s*_2_, ... *s*_*N*_} that are localized to the same location *L*, a background set of *N *sequences composed of proteins that are not localized to location *L *are selected. Identification of sorting motifs can thus be mapped to finding a motif model which can differentiate the motif instances from the positive sequence set from background sequences. The higher the classification accuracy of a motif model to differentiate the positive set from the negative set, the better the motif model.

We are interested in a special category of protein sorting motifs that are composed of a highly conserved, but short anchor and a comparably low-conserved motif region around the anchor. Usually these anchors have fewer than 4 amino acids, e.g.: in RR translocation pathway, the signal peptides all have a twin-arginine pair located between N and H region. And for LDL receptors, an NPXY motif frequently shows up at COOH terminal of the sequence. Because most of the sorting motifs are not well conserved at the amino acid level, it is thus difficult to find out these motifs by sequence alignment. Our approach is to firstly search the most frequent anchors in the positive dataset, and then use Naïve Bayesian classifier or other classifiers such as Support Vector Machines (SVM) to test if an anchor has a motif region around it that can differentiate them from background sequences (negative dataset). Our method is able to determine motif boundary using a sliding-window test to check classification accuracy of the subsequences in the windows.

**Figure 2:**
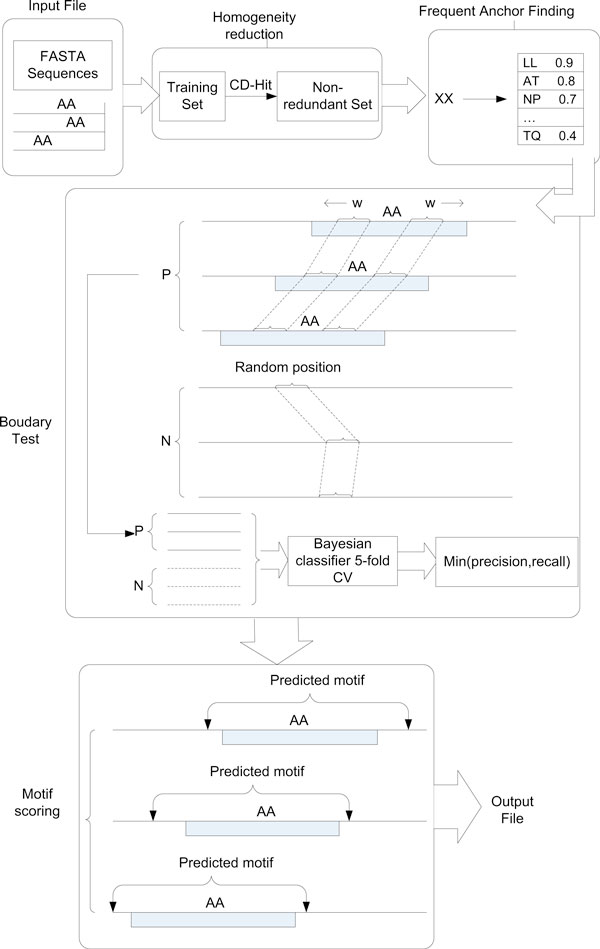
**Structure of BayesMotif algorithm for anchored sorting motif discovery**.

The BayesMotif discovery algorithm is composed of three major steps (Figure 2):

1) Preprocessing protein sequences by extracting K N-terminals and C-terminals amino acids and then applying sequence redundancy reduction using CD-HIT

2) Finding frequent anchors using regular expression enumeration;

3) Apply false positive removal to purify the positive training sequences

4) Constructing Bayesian classifiers to detect conserved motif regions around the anchors;

5) Based on the motif boundary given by step 3, calculate discrimination score for each motif using cross-validation test on Bayesian classifiers.

### Preprocessing of datasets

In the sorting motif discovery problem, a given set of proteins assumed to be transported to a specific location are given. These proteins can be either obtained from gene ontology annotation, genome scale localization experiments, or localization databases [3]. We also need to extract a set of background sequences from proteins which are not targeting that specific location. For each such sequence, we extract 200 amino acids from the N-terminal and C-terminal and apply the motif discovery algorithm on them.

An important preprocessing step is to remove redundancy in the training sequences. The rationale is that redundant training samples will cause classifiers to be biased to over-represented classes composed of redundant samples. Redundant training data will also fail the cross-validation testing lead to misleading prediction accuracy. To reduce the redundancy in the dataset, we use CD-HIT [15], a sequence clustering algorithm. It works by clustering the sequences by predefined or user defined weight matrices and a similarity threshold, and then removing all identical sequences in the same cluster but the pivot. It thus guarantees each pair of sequences in those left pivots will not be similar to each other. To make sure all sequences are not identical, the threshold for redundancy reduction is set to 80% in our experiments, which means the percentage of identical positions for two aligned sequences is less than 80%.

### Frequent anchor discovery

Frequent anchors are identified using exhaustive regular expression searching on the positive dataset. The search space is defined on a gap-tolerant regular expression anchor model since many protein sorting motifs (e.g. NPXY and YXXф motif in LDL receptors) are not completely conserved amino acid sequences, but a combination of two motifs with a variable-length gap. To find out these more flexible anchors, we use a regular expression model with the form: <Amino Acid>{n}<X>{min, max}<Amino Acid>{m} to represent the "language" of possible anchors. The anchor model is composed of two informative regions <Amino Acid>{n} and <Amino Acid>{m} with length n and m and a gap between these two regions <X>{min, max}. Here, min and max define the minimum and maximum gap length. We also allow the two motif regions to have defined length ranges and allow them to have different amino acid alphabets. Using this regular expression model, we can then enumerate all possible anchors and count their occurrence frequency in the positive dataset in both N and C terminal regions. We then check if there are conserved regions around these anchors and how these regions can differentiates the positive dataset from negative one.

### False positive removal

**Figure 3:**
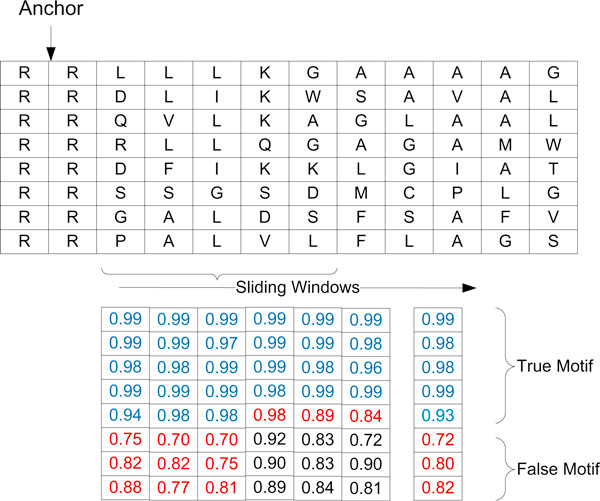
**False positive removal procedure**. This example shows BayesMotif uses sliding windows to remove false motifs from the dataset. It works by averaging the score before consecutive 3 window score which has at least one less than 0.85 (marked as red) calculate the score for each sequence the sequences have score less than 0.85 will be removed from the training set.

For each frequent anchor, we use the following iterative procedure to remove the sequences with false (non-motif) anchors from positive training samples (Figure 3)

1. Let a sliding window sit at anchor position for each sequence, extract the amino acids in the window as positive training samples for the Bayes classifier. For sequences without anchor, randomly place the window and extract subsequence within the window as negative training samples.

2. Train a naïve Bayes classifier for the current sliding window3. Score each sequence by calculating the probability-the window score, of a subsequence in ccurrent sliding window drawn from the trained Bayesian model4. Move the sliding window one amino acid to the left/right and go to step (2) until for all sequences, consecutive 3 window scores are all less than 0.85

5. For each sequence, calculate the average score for all windows, if it's less than 0.85, remove the sequence from the positive dataset

6. If no sequence is deleted in step (5) or the loop has reached a specified number, quit, otherwise go to step (1)

### Motif boundary determination

After generating the ranked anchor list and purifying training sequences, naïve Bayesian classifiers are trained to identify the most likely boundaries of the conserved regions around the anchors. For each anchor occurrence at N or C terminal in the positive dataset, BayesMotif uses a window of length W to slide from the anchor to the left, each time using the amino acids in the window as positive sequences for classifier training. For negative datasets, a randomly picked window within N or C terminal is used for extracting background training samples. After training a naïve Bayesian classifier, five-cross-validation is used to obtain the prediction accuracy of the classifier for a given sliding window. If the smaller value of precision and recall rates is lower than a given threshold (e.g. 0.5), it indicates that the sliding window has moved out of the true motif region and the left and right boundaries can thus be determined. It is obvious that the farther the sliding window leaves the motif, the more irrelevant regions will be included in the window, so the the score becomes lower. Similarly, the right boundary can be determined.

### Motif Score: measuring motif discrimination capability

After the left and right boundaries for an anchor are determined, we extracted the sub-sequences between the boundaries for all positive sequences and trained a naïve Bayesian classifier to obtain the overall classification score, which reflects the capability of the motif to differentiate the positive dataset from the negative dataset. This motif score is defined as min (precision, recall) to avoid the pitfall of unbalanced datasets.

### Motif information content: measuring motif conservation

We use the information content measurement [16] to measure the conservation level of discovered sorting motifs,: for a motif model of fixed length, each position in the model can be regarded as a random variable, the entropy of this random variable can be calculated according to Shannon theorem. Let *S *be the sequence set of a motif, the information content of an amino acid sorting motif can be calculated as: , where *p*_*j*_(*i*) is the frequency of amino acid *i*appearing in *j*th position of the sequence set; *A *is the amino acid alphabet; *L *is the motif length.

## Results and discussion

### Experimental setup

**Figure 4:**
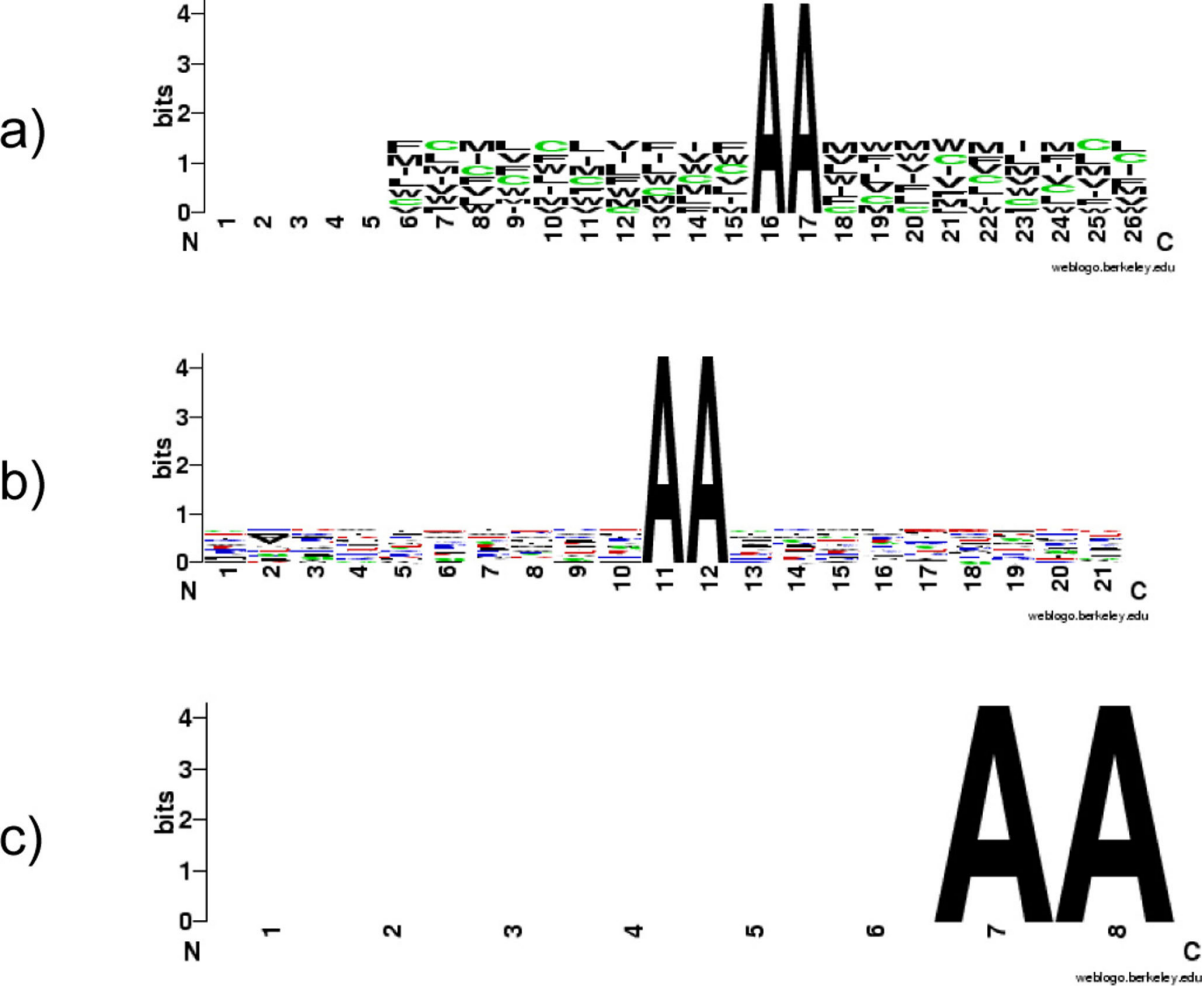
**Implanted motifs**. a) Simulated hydrophobic motifs anchored by AA; b) Simulated hydrophobic/positively charged motifs anchored by AA; c) No conserved motifs around anchors.

To evaluate our algorithm, we use both synthetic datasets and real datasets from Swiss-Prot release 48. Synthetic datasets are generated by inserting artificial motifs randomly in a set of protein sequences. Firstly, we chose the set of animal cytoplasmic proteins from Bacello dataset [9,17]) and applied the 80% redundancy reduction to get reduced Bacello dataset with 439 sequences. Next, we divided this sequence set into 219 positive and 220 negative sequences, used for implanting artificial motifs and as background sequences respectively. An artificial motif (in our experiments is----AA----) is inserted into a random position in the first 100 amino acids at the N-terminal of each sequence in the positive set. The random motifs are composed of a 2-amino acid anchor and a neighboring segment composed of amino acids drawn from an amino acid subset such as hydrophobic proteins {V, I, L, M, F, W, C}. The frequency of amino acids in the set is defined by a probabilistic distribution sampled from background dataset. This procedure allows us to simulate hydrophobic, charged or other physichemical regions typically occurring with the anchors. We use cytoplasmic proteins for both positive and negative datasets in order to guarantee they share identical background distribution of amino acids. Three sets of motifs are implanted into three positive datasets (Figure 4).

We used two real protein datasets containing experimentially verified motifs: the RR translocation signal peptide and the LDL receptors. We used the 439 animal cytoplasmic proteins of reduced Bacello dataset as negative protein sets. For real datasets, Tat-pathway translocation proteins and LDL receptors are extracted from Swiss Prot as shown in Table 1.

**Table 1:** Synthetic motif datasets and real datasets

Dataset	Number of positive samples	Number of negative samples	Anchors
Synthetic	219	220	----AA---- (Artificial)
Translocation	86	439	----RR----
LDL receptor	464	439	----NPXY----

### Results on synthetic datasets

We tested BayesMotif algorithm with three artificial motifs, each composed of the AA anchor and a less-conserved motif region of hydrophobic, hydrophobic+charged, and random amino acids. For each implanted motif, we randomly generate 10 sets of datasets. Each dataset has 219 positive and 220 negative samples. We compared the identified motifs with implanted ones in terms of their lengths, motif information content, and motif classification performance scores.

Table 2 shows that the basic BayesMotif without false positive removal has the capability to identify boundaries of the implanted anchored low-conserved artificial motifs. For the two artificial motifs with hydrophobic and hydrophobic+charged regions, the algorithm identified the implanted regions with motif scores of 1.0 and 0.98, which means that these two regions can differentiate positive sequences from negative almost perfectly with a minimum precision or recall rate of 1.0 and 0.98. We found that the information contents of the these two predicted motifs are higher than that of the random implanted motifs. It was also observed that the detected motif lengths are larger than that of the implanted motifs by 7 amino acids or 3-4 amino acids on both sides. This boundary errors can be reduced by tuning the classification accuracy threshold for boundary determination.

**Table 2:** Performance of BayesMotif for identifying implanted motifs

Motif	Implanted Motif length	Detected motif length	Information content	Motif Score
Hydrophobic	20	27.6 ± 2.4	33.1 ± 0.72	1.00 ± 0.0
Hydrophobic+Charged	20	27. 5 ± 3.0	27.9 ± 0 93	0. 98 ± 0.006
Random	20	25.4 ± 3.6	25.4 ± 0.85	0.79 ± 0.03

To test the effectiveness of the proposed false positive removal procedure, we generated a series of datasets with implanted motifs enriched with hyrophobic and charged regions around a 2-amino acid anchor. The positive set are implanted with a given percentage (from 10% to 100%) of artificial motifs. For each percentage, 10 datasets are generated and tested. The results are shown in Table 3 for basic BayesMotif algorithm and Table 4 for BayesMotif algorithm with false positive removal. From Table 3, it is observed that the basic algorithm can find implanted motifs with a motif score of 0.98 when all positive sequences contain the implanted motifs. When the percentage of true positive sequences decreases, the quality of the best predicted motif keeps decreasing with a motif score of only 0.55 for 10% positive rate, which has no overlap with the implanted motifs. The average motif information content decreases from 28 to 15. 9. Table 4 shows that BayesMotif with false positive removal can effectively address the impure dataset issue. The average motif information for the predicted motifs is pretty stable ranging from 27.9 to 23.9 when the percentage of true positive rate decreases from 100% to 20%. The motif classification scores are also much higher than that discovered by the basic BayesMotif. The enhanced BayesMotif identified motifs with scores greater than 0.90 when the true positive rate is larger than 40% compared to 0.59 to 0.80 of BayesMotif without false positive removal for positive rate from 40% to 80%. This means that the false positive removal procedure can help BayesMotif to identiy more conserved motifs with higher classification accuracy.

**Table 3:** Benchmark results of BayesMotif without false positive removal. Data are artificially generated by simulating hydrophobic and charged regions around a fixed 2 amino acids long anchor.

True motif Ratio	10%	20%	40%	60%	80%	100%
Motif Length	26 ± 3.24	26 ± 3.20	26 ± 4.1	29 ± 1.13	29 ± 1.8	27 ± 3.0
Motif Score	0.55 ± 0.04	0.55 ± 0.02	0.59 ± 0.01	0.67 ± 0.02	0.80 ± 0.01	0.98 ± 0.006
Motif Information	15.9 ± 0.87	15.3 ± 0.94	15.5 ± 1.3	17.7 ± 0.43	21.5 ± 0.62	28.0 ± 0.93

**Table 4:** Benchmark results of BayesMotif with false positive removal. Data are artificially generated by simulating hydrophobic and charged regions around a fixed 2 amino acids long anchor.

True motif Ratio	10%	20%	40%	60%	80%	100%
Motif Length	6 ± 0.83	17 ± 3.6	23 ± 1.2	23 ± 0.8	24 ± 0.7	27 ± 3.0
Motif Score	0.56 ± 0.11	0.79 ± 0.04	0.90 ± 0.01	0.95 ± 0.01	0.97 ± 0.007	0.98 ± 0.005
Motif Information	16.0 ± 1.1	23.9 ± 3.5	26.2 ± 1.2	26.3 ± 0.78	26.8 ± 0.39	27.9 ± 0.95

### Results on real datasets by BayesMotif

#### De-novo discovery of RR translocation signal peptide RR-x-FLK

**Figure 5:**
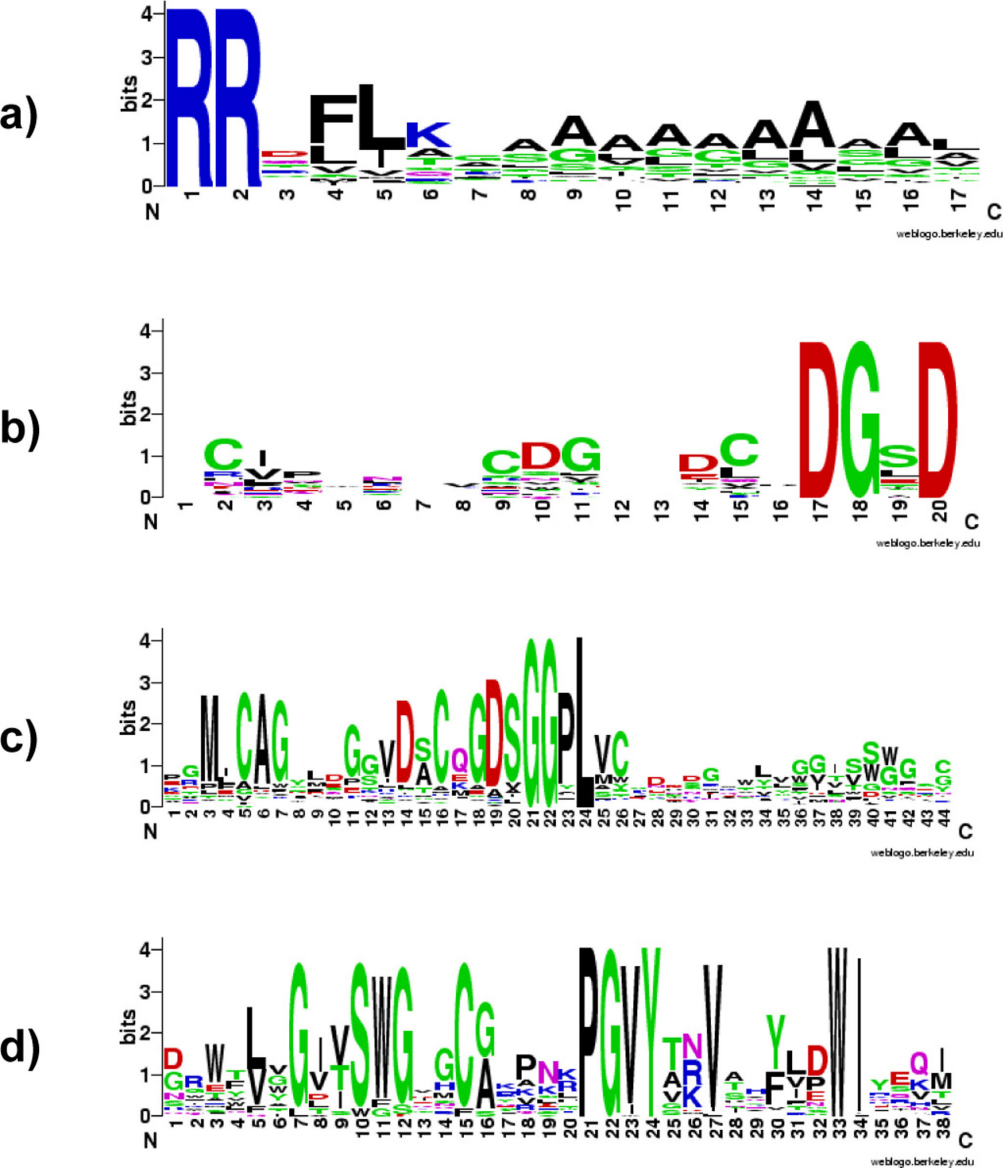
**Protein sorting motifs identified by the BayesMotif algorithm**. a) Motif logo of TAT-Translocation signal peptide RRxFLK; b) motif logo of DGxD motif; c) motif log of GGPL and GDSG motif; d) motif log of putative Motif PGVY.

TAT system is known as Sec-independent protein export pathway in bacteria. The most remarkable feature in TAT translocation proteins is the presence of the double arginines located between N and H region of the signal peptide. We downloaded 86 Tat-translocation proteins from SwissProt database and applied our BayesMotif algorithm with a two-amino acid XX anchor model. A set of 439 cytoplasmic proteins are used as the negative dataset. After homogeneity reduction with CD-Hit, our BayesMotif algorithm found the following motif with 17 amino acids (Figure 5a). The motif score is 87. 9, which means that the classifier can achieve classification accuracy of at least 0.879 in precision or recall rates. Although a functional RR-consensus motif RR-X-FLK is indispensable for targeting the Tat translocase, additional sequence features of RR-signal sequences seem to be required to prevent mistargeting to the Sec export pathway [18].

#### De-novo discovery of RR signal peptide RR-x-FLK with impure dataset

We also tested if BayesMotif with false positive removal algorithm can help to identify motifs from impure datasets. We picked 100 animal cytoplasmic proteins as negative dataset, and then selected 10, 20, ...80 Tat-translocation proteins matched with 90, 80, ...20 animal cytoplastic proteins as false positive proteins. Together we generated six datasets each comprising different percentages of true positive sequences. Then we run BayesMotif with or without false positive removal procedure on the datasets. We repeated 10 times of above procedure and the results are summarized in Table 5.

**Table 5:** Results of BayesMotif with or without false positive removal on real datasets

	True motif Ratio	10%	20%	40%	50%	80%	100%
Without False Positive Removal	Found by Algorithm	No	No	No	Yes	Yes	Yes
	Motif Score				0.55 ± 0.03	0.77 ± 0.01	0.91 ± 0.02
	Motif Information				19.0 ± 3.24	31.6 ± 0.5	39.5 ± 0.20
With False Positive Removal	Found by Algorithm	No	Yes	Yes	Yes	Yes	Yes
	Motif Score		0.63 ± 0.11	0.82 ± 0.04	0.83 ± 0.04	0.90 ± 0.02	0.92 ± 0.01
	Motif Information		21.5 ± 1.3	36.4 ± 2.4	38.0 ± 2.4	41.2 ± 1.1	42.0 ± 0.49

It is shown that BayesMotif without false positive removal cannot identify the known motifs when the positive rate is smaller than 40% while with false positive removal, BayesMotif can identify motifs if only the positive rate is equal to or larger than 20%. For the same positive rate equal or larger than 50%, the predicted motifs by BayesMotif with false positive removal have much higher information contents and also much higher motif scores--the classification performance to differentiate positive sequences from negative ones. For example, with positive rate of 50%, the new BayesMotif identified motifs with an average motif score of 0.83 compared to 0.55 for basic BayesMotif algorithm. The average information content is 38 compared to 19.

#### De-novo discovery of NPxY motif at C terminal of Megalin LDL receptor

Megalin is the main endocytic receptor of the proximal tubule and is responsible for reabsorption of many filtered proteins. It is found that information that directs apical sorting is present in the cytoplasmic tail (CT) of megalin, which contains three NPXY motifs, YXXØ, SH3, and dileucine motifs, and a PDZ-binding motif at its COOH terminus. Using 464 megalin sequences downloaded from Swiss-prot database as positive dataset and 439 animal cytoplasmic proteins as negative dataset, BayesMotif algorithm found the NPxY motif at the C-terminal along with a conserved amino acid region with undiscovered biological functionality (Figure 5b).

Besides the NPxY motif, we also found two other biologically verified motifs: DGxD motif and GGPL motif (Figure 5c, Figure 5d). DGxG motif is found in the alignment of five ligand-binding repeats in rat LRP3 protein. GGPL motif not only appears in LDL receptors but also in other protein families as GRF1-4 and OsGRF1, which presents as a C-terminal motif essentially related to transactivition activity [7].

BayesMotif also identified two additional motifs with significant high scores: GDSG and PGVY motifs. GDSG motif (Figure 5a) has a long motif region overlapped with GGPL motif, implying that it could work as a functional part of GGPL motif. PGVY(Figure 5d) is a new independent motif which has a well conserved motif region. The biological interpretation of this motif is still unknown, but significance from both frequency counting, information content, and discrimination scoring suggests that this motif is unlikely to be coming from random permutation of amino acids but instead has unknown biological significance.

### Comparison with other motif algorithms

We compared BayesMotif with two other popular protein motif discovery algorithms: MEME [6] and Teiresias [19]. MEME uses Position Weighted Matrix as motif models and searches overrepresented patterns on a given dataset by maximizing the motif likelihood using an EM algorithm. Teiresias uses a regular expression based frequent pattern mining algorithm for motif discovery.

We tested these two algorithms on the simulated datasets. It was found that Teiresias cannot retrieve any of the implanted motifs due to its inability to identify long motifs. MEME can find the implanted motifs but reported them as two separate motifs. We then tested the two algorithms on the real datasets and found that MEME and Teiresias can identify the following motifs RR-FLK, GGPL, and PGVY. But MEME failed to find the NPxY, GDSG, and DGxD motifs while Teiresias failed to find NPxP and DGxD motifs. However, both MEME and Teiresia tend to find short motifs while most protein sorting signals are composed of a short anchor and a region with less-conservation, which poses difficulty for such conventional algorithms. Another advantage of BayesMotif is that can work on very large datasets while current algorithms may not handle. Compared to conventional motif discovery algorithms, the classification based formulation of BayesMotif for motif discovery makes it easy to incorporate additional meta-sequence features for motif discovery such as hydrophobic or secondary structures and etc.

## Conclusion

We proposed BayesMotif, a Bayesian classifier based de novo protein motif discovery algorithm for identification of anchored protein sorting motifs. Experiments on both simulated datasets and real datasets demonstrated that the proposed algorithm is able to retrieve implanted anchored sorting motifs and identify experimentally verified sorting motifs. The proposed false positive removal procedure makes it possible to identify anchored sorting motifs even when the input data is not pure. It can help identiy more conserved motifs with higher classification performance. The web server for this program will be made available on the website of Machine Learning and Evolution Laboratory at University of South Carolina.

## Competing interests

The authors declare that they have no competing interests.

## Authors' contributions

J.H. proposed the research. J.H and F.Z proposed the algorithm. F. Z. implemented the algorithm. J.H. and F.Z. performed the experiments and analyzed the data. Both wrote and approved the manuscript.
